# Collectanea: Miscellaneous

**Published:** 1835-01-01

**Authors:** 


### MISCELLANEOUS.

### CHARITABLE ESTABLISHMENTS AT FLORENCE.

There are in Florence two large hospitals, and a Foundling hospital, where between two and three thousand infants are received annually. Scarcely any, however, are kept in the hospital; they are mostly put out to nurse in the country, and are supported by the establishment for the first ten years, and after that period otherwise provided for. The manner in which infants are swaddled up in cloths, something like an Egyptian mummy, the head only being left to move freely, is productive of frequent distortions of the limbs, and other bad effects.

The hospitals here, as elsewhere upon the continent, are superintended by the government, and patients are admitted on application, without any other recommendation than that of their requiring professional assistance. The Spedale Santa Maria Nuova is a handsome building, containing eight hundred beds. The wards are clean, spacious, and well ventilated. The professional treatment of the sick is in general judicious, and somewhat similar to the practice followed in France.

The Spedale di Bonifazio contains about the same number of beds as the other hospital, and is divided into two parts; one being for the insane, the other for persons afflicted with incurable diseases, and military men.

The average number of insane in the hospital is about three hundred. Small cells, having each a window with iron bars, and containing a bed, are placed upon either side of passages about fifty feet long. Each patient has a cell; but, in the day time, they are allowed to walk about the passages, and in open court-yards, and are all clothed alike, in a white woollen dress. The greatest attention is paid to cleanliness throughout the establishment; and patients, on their first arrival, are placed in separate rooms, in order that the peculiarities of their insanity may be observed by the physician. When confinement of the hands is necessary, a wooden case (manchôt) is used, into which both hands are placed, and confined, by means of a strap passing round the waist. Furious patients are confined singly in a small darkened room, well padded round the walls. The darkness and solitude are generally found to render them tractable. Moral means are adopted in the treatment; many of the patients being employed in mechanical occupations, or gardening; the women in knitting, spinning, &c. The system formerly in use of indiscriminately employing depletory measures every summer is now discontinued.

In the Casa dei Poveri, the poor are taught to work at various occupations. Those who, from age or other causes, are unable to work, are maintained by the establishment. Manufactures of cloth, carpeting, articles of clothing, &c. are carried on within the building.

The Societâ della Misericordia is another charitable establishment, instituted in the beginning of the fifteenth century, at the time of the great plague, and counts several of the nobility among its members. Its object is to render assistance to the sick poor, for whom the members perform many kind offices, and supply them with the necessaries of which they may stand in need. The society also undertakes the burial of the bodies of poor persons; and, in cases of accident, send to the spot where assistance is required, to convey the person to the hospital, or to his own residence. The brethren meet in a building in the Piazzo del Duomo, where their affairs are conducted by a committee. One or more members of the committee are always in attendance at the central institution, to indicate to the brethren on duty the place where their services are required. The sick are carried, in covered litters, on the shoulders of the brethren, who maintain a profound silence, and are clothed from head to foot in a black domino, in order to conceal the persons of those thus engaged, who are sometimes members of some of the first families in Florence. Ten, twelve, and often more brethren accompany each litter, and relieve each other in supporting the burden. There is a branch of this establishment at Pisa.—*Notes on Italy and Rhenish Germany, by Edwin Lee, Esq. M.R.C.S.*

### CAUSE OF THE COLOURS OF PLANTS.

Nothing can be named in the whole range of botany upon which information is so much wanted as the cause of the various colours of plants. It was indeed long since suspected by Lamarck that the autumnal colouring of leaves and fruits was a morbid condition of those parts, and it has subsequently been ascertained that all colours are owing to the presence of a substance, called chromule by De Candolle, which fills the parenchyma, assuming different tints. Green has also been clearly made out to be connected with exposure to light, and has been considered to be in all probability owing to the deposition of the carbon left upon the decomposition of carbonic acid. Some botanists have also observed the connexion of red colour with acidity; but still we had scarcely any positive knowledge of the cause of the production of any colour except green, till M. Macaire of Geneva remarked, that, just before leaves begin to change colour in the autumn, they cease parting with oxygen in the day, although they go on absorbing it at night; whence he concluded that their chromule is oxygenated, by which a yellow colour is first caused, and then a red, for he found that in all cases a change to red is preceded by a change to yellow. He also ascertained that the chromule of the red bractæ and calyx of *Salvia splendens* is chemically the same as that of autumnal leaves. Coupling this with the fact that petals do not part with oxygen, it would seem as if their colour, if yellow or red, may also be owing to a kind of oxygenation. But, according to M. Theodore de Saussure, coloured fruits part with their oxygen; so that, if this be true, red and yellow cannot always be ascribed to such a cause. M. De Candolle has some excellent observations upon this subject, in his recent admirable digest of the laws of vegetable physiology, in which he concludes, from the inquiries hitherto instituted, that all colours depend upon the degree of oxygenation. When oxygen is in excess, the colour seems to tend to yellow or red; and when it is deficient, or when the chromule is more carbonized, which is the same thing, it has a tendency to blue. Local additions of alkaline matters are also called in aid of an explanation of the various shades of colour that flowers and fruits present.

Dr. Dutrochet is of opinion that the whitish spots we sometimes see in leaves, and the paler tint that generally characterizes the under side of the same organs, are owing to the presence of air beneath the cuticle. He finds that the arrow-head shaped blotch on the upper side of the leaf of *Trifolium pratense*, and the whitish spots on *Pulmonaria officinalis*, disappear when the leaves are plunged in water beneath the exhausted receiver of the air-pump, and that the lower surface of leaves acquires the same depth of colour as the upper, under similar circumstances. This he ascribes to the air naturally found in the leaves being abstracted, and its place supplied with water; a conclusion which agrees with what might be inferred from the anatomical structure of the parts in question.—*Professor Lindley, in the Third Report of the British Association for the Advancement of Science.*

EXPERIMENTS ON ATOMIC WEIGHTS. BY DR. TURNER.

Dr. Turner reported to the Meeting that he had continued his researches into atomic weights, and had to his own conviction determined the points which had induced him to undertake the inquiry. These were, first, to form an opinion of the relative accuracy of the tables of equivalents employed in this country and on the continent; and, secondly, to ascertain whether there existed any trustworthy evidence in proof of the hypothesis that the equivalents of bodies are multiples by whole numbers of the equivalent of hydrogen. To examine these questions he endeavoured to ascertain by careful and often-repeated experiments the equivalents of silver, chlorine, lead, barium, mercury, and nitrogen, in relation to oxygen. These were selected in consequence of their frequent use in analysis. An error in these could not exist without affecting the equivalents of nearly all the other elementary substances. The researches on this subject had been lately read before the Royal Society, and would probably ere long be published in some form or other. The general result is, that the atomic weights current in this country are much less exact than those given by Berzelius; that though they had been recommended to British chemists as rigidly correct, they were often very inexact, and had been determined by methods which in some important cases were defective. Further, he finds that, as far as experimental evidence at present goes, the hypothesis above alluded to is unsupported. In some instances the equivalents are so nearly simple multiples of that of hydrogen that they may be taken as such without appreciable error; but in many other cases the numbers given by experiment cannot be reconciled with the hypothesis. The following are the numbers which he is disposed to believe very nearly correct: lead 103.6; silver 108; chlorine, 35.42; barium, 68.7; mercury, 202, perhaps slightly higher, but not higher than 202.3: nitrogen, 14.2. Dr. Turner states that his methods for ascertaining nitrogen were not so advisable as that in which Dr. Prout is occupied by weighing the gases. This weight should be kept in abeyance for the present. He conceives that it does not fall below fourteen, nor exceed 14.2. During these researches he incidentally obtained some facts for inferring the equivalent of silver; and from these it appears that the equivalent of sulphur is nearer 16.1 than 16. He would not venture, however, to make a positive statement without further inquiry.

He then mentioned that Dr. Prout had kindly informed him of a fact which he conceived analytical chemists in general to be ignorant of, and which he thought might have had an influence on these researches. The fact is, that chloride of silver, however white and well washed, gives out a little muriatic acid at the moment of fusing. This fact Dr. Turner has examined, and can confirm. It especially ensues when fusion takes place before the chloride has been well dried; but, in the event of the chloride of silver being first well dried at 300° (when no acid is given out,) and then, without exposure to the atmosphere while cold, fused, the loss of acid is not appreciable in weight, though it is still sufficient to redden delicate litmus paper. In two experiments about fifty grains of chloride of silver were fused, (previously dried at 300°, introduced while hot into a dry bottle furnished with a tight cork, and weighed in that state,) and the loss was inappreciable. From this circumstance, taken in conjunction with the mode in which he habitually weighs the chloride of silver, he is satisfied that the fact observed by Dr. Prout does not necessarily produce any error in the determination of chlorine by means of silver.—*Third Report of the British Association.*

### VEGETATION NEAR QUITO.

It seems more easy to naturalize the vegetable productions of Europe in the regions of the Andes, than *vice versa*. European flowers adorn the gardens, and European vegetables supply the tables of Quito, as of every part of the table lands. The introduction of the *Cerealia* is one of the few benefits conferred by the Spaniards on the New World. The Indigenes appear to have used only *Maize*, the *Chenopodium Quinua*, the *Potato*, and the *Oxalis tuberosa*, the *Oka*. Barley meal constitutes at present the chief article of their diet; for bread, though cheap, scarcely falls within their scanty resources. Oats and rye are as yet unknown, though well adapted to many of the poorer soils, especially the sandy tracts round Ambato and Riobamba. The same cause which prevents the perfection of European fruit limits the number of those of native growth; about the elevation of Quito, we find none wild but the *Capuli*, a species of *Blackberry*, and, on sandy soils, the *Tuna;* currants, gooseberries, and raspberries, seem adapted to the climate, but have not yet been introduced. Strawberries are abundant, but they are probably natives of Chili. Pears and apples are plentiful, but small and ill-flavored. The celebrated peaches of Ambato remind the European traveller less of the likeness than of the difference. *Pine Apples*, *Cherimoyas*, *Oranges*, *Limes*, *Aguacatis* (*Laurus Persea*), *Granadilla* (*Passiflora*—?) and other tropical fruits, are brought from the adjacent valleys or *Calientes;* but, it may be supposed, little improved by the journey.

The idea of *perpetual spring* is pleasing to the imagination, but the reality is purchased in the Andes by the want of those glowing forms and colours which nature sheds over tropical climates, while the monotony of earth and sky scarcely observable by the traveller, would be gladly exchanged, by the less fortunate resident, for the varied interest of European seasons.—*Colonel Hall, in Hooker's Journal of Botany.*

### THE PLATYSMA MYOIDES.

Resuming the subject, then, the first thing to which I beg your attention is a single muscle, the platysma myoides, and I venture to say, that so long as this muscle is viewed by the student only as Albinus or Cowper, or any other authority upon the anatomy of this part, represents it on paper, he remains ignorant of its functions, and scarcely understands that it is anything more than a cutaneous muscle, which in brutes, for example, is an aid for digging up insects and other food, whereas it is a powerful muscle both of respiration and circulation.

In a certain condition of the respiratory organs the blood experiences great difficulty in descending from the head, and nature, by the position and action of this very muscle, guards the head, and prevents the congestion which would otherwise take place. It arises broad on the side of the chest, passes over the veins, the great and external jugulars, and passes up to the face. In difficult respiration, you will observe the shoulder raised and brought back again; with the clavicle is raised the sterno-cleido-mastoideus muscle, and at the moment the clavicle is raised, and the sterno-cleido-mastoideus muscle is heaved up, as it were, this muscle is in a state of relaxation, at which period all the great veins, which might with much propriety be called sinuses, the large veins of the neck, fill easily with the blood which is returning from the head. And, again, when the action comes to be the very reverse, and more especially when this muscle is brought into action, then the blood does not return from the head; it is prevented from returning, but the muscle presses out the blood into the heart, even against the power of respiration.

Thus I conceive that nature is bountiful and careful to this end, that in all natural postures of the body, the brain shall not sustain that injury which it often would do, if there were no particular provision against congestion of the vessels which convey the blood away from the brain. Accordingly, in powerful exertion, during a fit of coughing, or at any other time when the respiration is powerfully occupied, you will see that this muscle acts most beneficially; and you will further observe, that in attacks of asthma, where a difficulty of respiration is frequent, where there are returns of pain and excitement greater each time, this muscle may, like all other muscles of the body, become proportionably strong and powerful as it is oftener employed. There is, in fact, a drawing down of the angle of the mouth, and a prominent character, acquired in asthmatic persons by the action of this muscle.—*Sir C. Bell's Lectures.—Lancet.*

MORTALITY FROM PHTHISIS AT DIFFERENT AGES.

| | *Place of observation.* | 15 to 20 | 20 to 25 | 25 to 30 | 30 to 35 | 35 to 40 | 40 to 45 | 45 to 50 | 50 to 55 | 55 to 60 | Above 60 |
|---|---|---|---|---|---|---|---|---|---|---|---|
| 1 | Edinburgh .......... | 6 | 9 | 13 | 8 | 11 | 8 | 6 | 3 | 9 | 4 |
| 2 | Berlin .................. | 18 | 28 | 27 | 27 | 39 | 29 | 20 | 32 | 39 | 53 |
| 3 | Nottingham............ | 42 | 73 | 76 | 46 | 51 | 28 | 20 | 11 | 5 | 6 |
| 4 | Philadelphia .......... | 182 | 974 | | 875 | | 565 | | 338 | | 258 |
| 5 | Chester .................. | 15 | 27 | | 24 | | 22 | | 16 | | 6 |
| 6 | Carlisle .................. | 15 | 45 | | 34 | | 31 | | 15 | | 15 |
| 7 | Paris (*Louis*)......... | 11 | 39 | | 33 | | 23 | | 12 | | 5 |
| 8 | Ditto (*Bayle*)......... | 10 | 23 | | 23 | | 21 | | 15 | | 8 |
| 9 | Charleston ............ | | 26 | | 24 | | 13 | | 21 | | 4 |
| 10 | Ditto, Whites......... | | 14 | | 17 | | 10 | | 3 | | 3 |
| 11 | Ditto, Blacks ......... | | 15 | | 13 | | 9 | | 3 | | 3 |

*Dr. Clark, in Cyclop. of Pract. Med.* Part xxii.

EXCRETIONS OF PLANTS.

It has long been known that some plants are incapable of growing, or at least of remaining in a healthy state, in soil in which the same species has previously been cultivated. For instance, a new apple orchard cannot be made to succeed on the site of an old apple orchard, unless some years intervene between the destruction of the one and the planting of the other: in gardens, no quantity of manure will enable one kind of fruit-tree to flourish on a spot from which another tree of the same species has been recently removed; and all farmers practically evince, by the rotation of their crops, their experience of the existence of this law.

Exhaustion of the soil is evidently not the cause of this, for abundant manuring will not supersede the necessity of the usual rotation. The celebrated Duhamel long ago remarked, that the elm parts by its roots with an unctuous dark-coloured substance; and, according to De Candolle, both Humboldt and Plenck suspected that some poisonous matter is secreted by roots; but it is to M. Macaire, who at the instance of the first of these three botanists undertook to inquire experimentally into the subject, that we owe the discovery of the suspicion above alluded to being well founded. He ascertained that all plants part with a kind of fæcal matter by their roots, that the nature of such excretions varies with species or large natural orders: in *Chicoraceæ* and *Papaveraceæ* he found that the matter was analogous to opium, and in *Leguminosæ* to gum; in *Gramineæ* it consists of alkaline and earthy alkalies and carbonates, and in *Euphorbiaceæ* of an acrid gum-resinous substance. These excretions are evidently thrown off by the roots on account of their presence in the system being deleterious; and it was found by experiment that plants artificially poisoned parted with the poisonous matter by their roots. For instance, a plant of *Mercurialis* had its roots divided into two parcels, of which one was immersed in the neck of a bottle filled with a weak solution of acetate of lead, and the other parcel was plunged into the neck of a corresponding bottle filled with pure water. In a few days the pure water had become sensibly impregnated with acetate of lead. This, coupled with the well known fact that plants, although they generate poisonous secretions, yet cannot absorb them by their roots without death, as for instance is the case with *Atropa Belladonna*, seems to prove that the necessity of the rotation of crops is more dependent upon the soil being poisoned than upon its being exhausted.—*Third Report of the British Association.*

### ENTRIES IN THE OLD BILLS OF MORTALITY.

One of the regular entries, two centuries ago, was " Chrisomes and infants;" thus substituting the age of the deceased for the disease. By *chrisome* was meant merely a child not yet a month old, the appellation being derived from the *chrisom*, or cloth anointed with holy unguent, which infants wore till they were christened. Graunt observes, that, as the number of deaths put down to this head decreased, the number set down to convulsions increased. The reason of this is quite obvious; the more diligent searchers, not satisfied with being told that an infant had died a chrisome, asked for the name of the disease, and convulsions are certainly the most fatal malady by which young infants are carried off. The following table will show the gradual decrease of deaths under this head:

| | Years. | Deaths. |
|---|---|---|
| Chrisomes and infants . . | 1657 | 1162 |
| — . . | 1678 | 301 |
| — . . | 1680 | 183 |
| — . . | 1688 | 209 |
| — . . | 1699 | 70 |

In the bill for 1701 the number is stated separately for the two subdivisions—

Chrisomes, 44; Infants, 29; making 73.

In 1702, we have, Chrisomes . . . . . . . 49

— Infants . . . . . . . 18

In 1726 there are but three chrisomes, being the last time this entry appears: " infants" occurred for the last time in 1722.

" Blasted and planet," is another very curious entry: we find five deaths under this head in 1657, five in 1658, three in 1659, and eight in 1660. " Planet" is then found no more, and " blasted" soon disappears. " Planet-struck," however, (of which " planet" seems to be an awkward abbreviation), occurs during the casualties for several years afterwards. These appellations were probably bestowed on persons who wasted away without any very obvious cause, and whose deaths would be attributed by physicians to

"marasmus or wide-wasting atrophy," but by the vulgar to the influence of some sinister planet. The Latin word *sideratus* implies a theory of the same kind.

Three deaths are set down to "Calenture" in 1657, three in 1673, one in 1681, and one in 1708. This is a disease, say the books, (for we have never seen a case ourselves,) in which sailors throw themselves into the sea, imagining it to be green fields. It would seem, therefore, to be a form of *nostalgia*, the *maladie du pays*, or longing for home, a malady capable of producing death among the enthusiastic inhabitants of mountainous countries. "Calenture" does not occur in the modern bills.—*The Companion to the Almanac; art. Bills of Mortality.*

### FALSIFICATION OF WRITINGS.

A medical student was recently convicted and punished in France, for forging a will, by altering the handwriting; and it is said that frauds of this nature are becoming alarmingly frequent in that country of late. We should not be surprised if similar attempts were made here, ere long, by some of our *chevaliers d'industrie;* but fortunately the mode of detecting falsifications of the kind is not difficult, if suspicion be once awakened. Old writing is removed, and place made for new, either by erasure, by the application of muriatic acid, or by both. A supposed erasure should be examined by a magnifier; and, if it seem to have been touched with size or sandarac, as a ground for the new writing, these substances may be dissolved with water or alcohol respectively. If muriatic acid have been used, it is not unlikely that all the acid may not have been washed out, when the excess may be readily tested with litmus paper. But, whether chlorine or muriatic acid have been employed, it is probable that some trace of the oxide of iron of the old ink may still remain; and these may be rendered visible by washing the part with a camel- hair pencil dipped in diluted gallic acid. The latter process may have to be repeated several times, and on successive days, allowing the dilute acid to evaporate slowly each time. We are indebted to a paper by M. Chevallier, in the last Annales d'Hygiène, for these brief but valuable suggestions.—*Med. Gazette.*

### ACCIDENTAL VACCINATION.

A practitioner in the neighbourhood of St. Mary Axe, who was a few days since called to vaccinate a child, met with the following accident. At the moment he was about to perform the operation, the little patient, by a sudden struggle, drove the hand of the surgeon towards his face, and made the lancet slightly penetrate his nose. The vaccine matter in a short time took effect on the practitioner's frontispiece, and so marked it with the eruption, that he has been compelled to leave his practice for a time, and seek a clean face in the country.—*Lancet.*

### THE DEATH OF HANNIBAL.

But what was the poison contained in that "Cannarum Vindex, et tanti sanguinis ultor *Annulus*," by which Hannibal destroyed himself? When the tyrant of Bithynia had pointed out to his enemies, who were in pursuit of him, the house in which Hannibal lodged, the unfortunate general, finding his fate inevitable, said, according to Livy, "Now* will we liberate these Romans from their unceasing solicitude about us: they are tired, it seems, of waiting for the death of an old man;" and took the poison. What it was it is almost impossible that we should ever know. Modern chemistry, indeed, could furnish twenty poisons capable of being comprehended within the space of a ring. One drop of *Prussic acid*, contained in a small glass tube open at both ends, and held between the finger and thumb, so as to touch both when in motion, would paralyze the arm almost instantaneously, and, of course, if taken into the stomach, would forthwith arrest the current of life. But, although the Carthaginians were a much more civilized people than their enemies the Romans (who happen to be their historians,) are willing to allow, yet it is too much to suppose that they knew how to prepare the Prussic acid. No, *Lybia ferax venenorum*, Lybia abounding in the venom of serpents, and in the inspissated juices of deleterious vegetables, more probably furnished them with the poisons in question, and afforded to Hannibal a sure resource whenever his circumstances should become desperate.†

As to the report of his being poisoned by drinking *bullock's blood*, mentioned by Plutarch, it must be a fable; as was that also of the death of Themistocles, by drinking a similar draught, for the blood of that animal is not poisonous. An accomplished nobleman told me that he was present at one of the bull-fights at Madrid, when a person rushed from the crowd, and, having made his way to the bull, which the matador had just stricken, caught the blood as it flowed from the wound in a goblet, and drank it off before the assembly. On inquiring into the object which the poor Spaniard had in view, it appeared that the blood of a bull just slain was a popular remedy for consumptive symptoms.—*Sir H. Halford's Essays. Deaths of Illustrious Persons of Antiquity.*

* Solvamus diuturnâ curâ populum Romanum, quando mortem senis expectare longum censet.—Livy.

† My friend Mr. Hatchett conjectures that the poison which Hannibal took might have been the inspissated exudation of the Euphorbia officinalis. The Euphorbia is a native of Africa, abundant there, and was well known as one of the most powerful acrid vegetable poisons.

